# Nano-BCG: A Promising Delivery System for Treatment of Human Bladder Cancer

**DOI:** 10.3389/fphar.2017.00977

**Published:** 2018-01-12

**Authors:** Julieti Huch Buss, Karine Rech Begnini, Camila Bonemann Bender, Adriana R. Pohlmann, Silvia S. Guterres, Tiago Collares, Fabiana Kömmling Seixas

**Affiliations:** ^1^Laboratory of Cancer Biotechnology, Biotechnology Graduate Program, Technology Development Center, Federal University of Pelotas, Pelotas, Rio Grande do Sul, Brazil; ^2^Pharmaceutical Sciences, Federal University of Rio Grande do Sul, Porto Alegre, RS, Brazil; Institute of Chemistry, Federal University of Rio Grande do Sul, Porto Alegre, Rio Grande do Sul, Brazil; ^3^Pharmaceutical Sciences, Federal University of Rio Grande do Sul, Porto Alegre, Rio Grande do Sul, Brazil

**Keywords:** bladder cancer, nanotechnology, BCG, Nano-BCG, monoclonal antibody, EGFR

## Abstract

*Mycobacterium bovis* bacillus Calmette–Guerin (BCG) remains at the forefront of immunotherapy for treating bladder cancer patients. However, the incidence of recurrence and progression to invasive cancer is commonly observed. There are no established effective intravesical therapies available for patients, whose tumors recur following BCG treatment, representing an important unmet clinical need. In addition, there are very limited options for patients who do not respond to or tolerate chemotherapy due to toxicities, resulting in poor overall treatment outcomes. Within this context, nanotechnology is an emergent and promising tool for: (1) controlling drug release for extended time frames, (2) combination therapies due to the ability to encapsulate multiple drugs simultaneously, (3) reducing systemic side effects, (4) increasing bioavailability, (5) and increasing the viability of various routes of administration. Moreover, bladder cancer is often characterized by high mutation rates and over expression of tumor antigens on the tumor cell surface. Therapeutic targeting of these biomolecules may be improved by nanotechnology strategies. In this mini-review, we discuss how nanotechnology can help overcome current obstacles in bladder cancer treatment, and how nanotechnology can facilitate combination chemotherapeutic and BCG immunotherapies for the treatment of non-muscle invasive urothelial bladder cancer.

## Introduction

Bladder cancer (BC) is the second most common malignancy of the urinary tract, the fourth most common cancer in men with a yearly incidence rate of 330, 380 cases, and the 11th most common among women with a yearly incidence rate of 99,413. Worldwide, carcinomas of the bladder represent the ninth most common cause of cancer, with 430,000 patients diagnosed with BC annually (Jemal et al., 1999; Ferlay et al., 2013). The incidence of BC also increases with advancing age, as 90% of new diagnoses are made in people over the age of 55 (average age of 73 years at diagnosis) (American Cancer Society, 2017).

Most (75%) BC cases are non-muscle-invasive bladder cancers (NMIBC) at diagnosis with the other 25% representing muscle invasive bladder cancers (MIBC) or metastatic cancers (Moch et al., 2016). Urothelial carcinomas can be categorized as low grade or high grade according to their architectural and cytological atypia and include papillary urothelial neoplasm or low malignant potential (Cheng et al., 2012). Pathological assessment is the gold standing for tumor classification. Ta (non-invasive papillary) and Tis [carcinoma *in situ* (CIS)] are tumors that are restricted to the mucosa, while T1 and T2 are tumors that invade the lamina propria and the muscularis propria, respectively (Sobin and Gospodarowicz, 2009).

Initial BC treatments involve transurethral resection (TURBT) to facilitate removal of the visible tumor (Hall et al., 2007; Sylvester, 2008; Babjuk et al., 2013). Further therapy is dependent on pathologic stage and grade of the tumor and often mediated through intravesical instillation. Although the response rate to therapy in patients with NMIBC is high (~80%), 50–90% of NMIBC patients suffer from recurrence within 5 years, with muscle invasion found in up to 20% of recurrent patients (Rübben et al., 1988; Lamm and Allaway, 2000; Hussain et al., 2009). This review focuses on currently available BC therapies and describes nanotechnology tools to enhance therapeutic effects and overcome side effects, emphasizing its use to improve BCG immunotherapy.

## Bladder cancer treatments

Following TURBT, a single intravesical chemotherapy treatment is recommended for patients with low to intermediate risk NMIBC, with mitomycin, epirubicin, and gemcitabine representing common drugs of choice (Kamat et al., 2016). It has been shown that the relative risk for tumor recurrence is reduced by 50% if the chemotherapy instillation is given with 24 h after TURBT (Kaasinen et al., 2002).

Intravesical immunotherapy with *Mycobacterium bovis* Bacillus Calmette-Guérin (BCG) is the treatment of choice for patients with high-risk NMIBC. BCG immunotherapy is the gold standard treatment for NMIBC due to its ability to reduce recurrence and progression to MIBC (Ahn et al., 2014). A meta-analysis with individual patient data comparing BCG immunotherapy with intravesical mitomycin chemotherapy has shown BCG to be superior in terms of reducing recurrence and delaying disease progression; however, no significant differences in progression or overall survival were observed (Malmström et al., 2009). Conventional BCG treatment consists of a percutaneous BCG vaccine administered 2–6 weeks after TURBT followed by 6 weekly courses of intravesical BCG administration (Morales et al., 1976; Kresowik, 2009; Kamat et al., 2015). However, specific BCG substrain preferences, schedules, and dosages differ across geographic regions due the wide range of BCG substrains licensed for human tuberculosis vaccination and BCG immunotherapy (Gan et al., 2013). Based on multiple meta-analyses, it is recommended to continue BCG therapy for 1 to 3 years if tolerated by the patient to decrease recurrence and progression of NMIBC (Shelley et al., 2001; Sylvester et al., 2002; Böhle and Bock, 2004; Hall et al., 2007; Gontero et al., 2010; Babjuk et al., 2013).

Despite these guidelines, it is estimated that 20% of patients with high-risk NMIBC treated with BCG will progress to muscle invasion or suffer from NMIBC recurrence within 5 years (Rübben et al., 1988). MIBC is a major clinical issue due to its aggressiveness and high 5 year mortality rate. To maximize survival rates, radical cystectomy (RC) represents the best treatment option. RC consists of removal of the bladder, prostate, seminal vesicles, proximal vas deferens, and proximal urethra in men, and bladder, uterus, ovaries, fallopian tubes, urethra, and part of vagina in women (Arcangeli et al., 2015). Many patients cannot tolerate the morbidity of RC and instead opt for continued local therapy as an effort to spare their bladder (Ahn et al., 2014). Some studies have shown progress with the use of Mytomicin C (van der Heijden et al., 2004; Halachmi et al., 2011), Gemcitabine (Skinner et al., 2013), Valrubicin (Steinberg et al., 2000), Docetaxel (Barlow et al., 2013), Nab-Paclitaxel (McKiernan et al., 2011), mycobacterial cell wall extract (Morales et al., 2009), EGFR (Rebouissou et al., 2014) and a combination therapy of Gemcitabine and Mytomicin C for the treatment of BC (Lightfoot et al., 2011). More recently, trimodal treatment with simultaneous delivery of chemotherapy and radiotherapy has emerged as an effective bladder sparing treatment with similar survival rates compared to RC (Arcangeli et al., 2015). However, none of these local therapies have shown to be more effective for BC control than early RC (Ahn et al., 2014).

## Chemotherapy optimization using nanotechnology for bladder cancer therapy

Nanotechnology consists in the study and application of materials on the nanometer scale (Ebbesen and Jensen, 2006) and application of nanotechnology in the medical field is referred to as nanomedicine (Sweeney, 2015). Nanotechnology have proven to be a powerful tool for the development of new chemotherapies or immunotherapies for BC. The development of new drug delivery systems has been growing and is expected to continue to increase over the next few years (Brito et al., 2017).

In this context, several studies have utilized nanoparticles (NPs) to increase the therapeutic effectiveness and reduce adverse effects of chemotherapy by targeting chemotherapeutic agents to a specific tissue and increasing its bioavailability (Yurgel et al., 2014; Kang et al., 2017; Yao et al., 2017; Zhu et al., 2017). Polysaccharide-based NPs loaded with Mitomycin C and surrounded by the bioadhesive polymer chitosan mixed with polylactic acid or with poly(ε-caprolactone) have been utilized in an attempt to optimize BC treatments. This NP promoted favorable drug loading and release profiles along with improved anticancer efficacy and cellular interactions (Bilensoy et al., 2009). Erdogar et al. (2012) has also demonstrated that bioadhesive and cationic NPs loaded with Mitomycin C are able to increased exposure of the bladder to the drug resulting in a drug reservoir at the action site, which might improve local treatment (Erdogar et al., 2012). In addition, cationic core-shell nanoparticles loaded with Mitomycin C have also improved antitumor efficacy in tumor-induced rat models (Erdogar et al., 2014).

Magnetic NPs (MNPs) also show promise for delivery of quimiotherapic agents to the target tissue (Stapf et al., 2017). To limit doxorubicin's (Dox) cytotoxic effects on healthy cells, MNPs (iron oxide) were conjugated with Dox to ensure efficient delivery to cancer sites, resulting in increased BC sensitivity compared to Dox alone (Nowicka et al., 2013). In addition, monoclonal antibodies (mABs) bound to MNPs can increase the ability of MNPs to target BC cells and enable thermotherapy to cope with BC recurrence (Rezaei et al., 2017).

NPs loaded with molecules with high urothelium mucoadhesivity is another approach used to optimize the delivery of molecules to the target tissue. To increase Dox specificity for BC cells, thiol-functionalized NPs loaded with Dox were synthesized and induced cytotoxicity against UMUC3 cancer cells (Zhang et al., 2014). Besides the possibility of conjugation with different molecules, studies have also reported the importance of developing methods to modify the surface of mesoporous silica NPs to enhance the antineoplasic effects of Dox on BC (Wei et al., 2017). An enhanced therapeutic effect against UMUC3 cells was also demonstrated using Dox and peptide-modified cisplatin synergistically loaded onto positively charged mucoadhesive chitosan–polymethacrylic acid nanocapsules (Lu et al., 2016).

In addition, platinum agents can be loaded onto a variety of polymeric, lipid, and inorganic nanocarriers, including liposomes, NPs, and nanotubes to increase their antitumoral effects (Browning et al., 2017). The use of cisplatin nanocarriers is associated with reduced toxicity and adverse events (Sudha et al., 2017); however, novel strategies are required to increase drug uptake and release at the target site. In this regard, cisplatin NPs were evaluated in a preclinical study against NMIBC and cisplatin-loaded biocompatible poly(L-aspartic acid sodium salt) (PAA) NPs demonstrated potential for improved intravesical treatment of NMIBC while reducing local and systemic side effect (Kates et al., 2017).

Nanotechnology tools have also been used in clinical trials. Albumin-bound-Paclitaxel NPs showed minimal toxicity and systemic absorption when used to treat NMIBC during the first human intravesical phase I trial (McKiernan et al., 2011). In addition, phase II trials have demonstrated minimal toxicity of intravesical nab-paclitaxel in NMIBC patients with a response rate of 35.7% (Mckiernan et al., 2014). The formulation of albumin-bound-paclitaxel NPs has also been used for the first time to treat unresectable metastatic urethral cancer. Follwoing therapy, a 70% reduction in the size of the tumor was observed in addition to 19 months of progression free survival (Abaza and Alemany, 2014).

## RNAi optimization of nanotechnology bladder cancer therapy

The use of interference RNA (RNAi) combined with nanotechnology is another promising approach for BC treatment. RNAi technology can be used to inhibit tumor growth through messenger RNA inhibition of several activated oncogenes (Xin et al., 2017). In human BC, some upregulated genes associated to the development of resistance to chemotherapy have been inhibited using RNAi technology through knockdown of the target gene (Pan et al., 2016; Wang et al., 2017). Within this context, RNAi technology is a highly effective approach to combat chemoresistance and improve advanced BC outcomes.

Although RNAi technology could be used to overcome multidrug resistance and restore cells sensitivity, there are several challenges associated with RNAi delivery to diseased sites for gene therapy (Melamed et al., 2017). NPs appear to be a promising tool to help overcome existing biological barriers to RNAi delivery. Through this approach, studies have reported upregulation of BC specific genes, which can be effective targets of NP-siRNA therapeutic approaches (Seth et al., 2011; Müller et al., 2016).

Small dsRNAs, known as small activating RNA (saRNA), produce the opposite effect of RNAi by inducing gene expression (Li et al., 2006; Chen et al., 2008). The therapeutic potential of dsRNA (P21-322) coupled with nanotechnology has been demonstrated using a 20-fluoro-modified derivative loaded into lipid NPs in an orthotopic model of BC. Antitumoral activity and induction of p21 expression was confirmed *in vitro* and *in vivo* (Kang et al., 2012), suggesting induction of specific genes can provide an alternative route to BC treatment.

## Functionalization of nanoparticles to optimize bladder cancer therapy

The functionalization of nanoparticles with monoclonal antibodies is a promising strategy for targeted delivery to and uptake by cells overexpressing the antigens specific for these antibodies (Eloy et al., 2017). Development of molecules that exhibit affinity for targets expressed on tumor cells represents an emerging therapeutic approach (Diesendruck and Benhar, 2017). For example, the receptor tyrosine kinase EGFR exhibits altered expression in several types of solid tumors and its overexpression in UC is directly correlated with advanced tumor stages (Kassouf et al., 2008). The incidence of EGFR positivity in epithelial tumors varies by tumor type. BC is often characterized by a high mutation rate and high EGFR expression in approximately 50% of cases (Colquhoun and Mellon, 2002). Thanks to the expression of EGRF on BC cells, an anti-EGFR monoclonal antibody conjugated to gold nanorods was able to effectively bind EGFR-expressing BC cells and reduce the systemic exposure and clearance of nanoparticles from the body (Yang et al., 2015). These results indicate the use of monoclonal antibodies represents another possible approach for delivery of molecules to specific tissues.

## Nano-BCG: optimizing BCG immunotherapy using nanotechnology to treat bladder cancer

As previously mentioned, BCG is considered the standard treatment for NMIBC. However, BCG immunotherapy is associated with frequent induction of adverse effects in patients (Poletajew et al., 2017) leading researchers to investigate novel alternatives to increase their effectiveness (Begnini et al., 2013). Delivery systems and nanotechnological approaches are interesting tools to improve currently available BCG therapies and prolong exposure of the bladder tissue. The main advances of nanotechnology tools for improvement of BCG immunotherapies against BC are listed in Table 1.

**Table 1:** Major studies involving nanotechnology tools applied to BCG immunotherapy against bladder cancer.

**Study objective**	**Approach used**	**Nanoparticle name**	**Preparation method**	**Mean nanoparticle size**	**Zeta potential (mv)**	**Entrapment efficiency**	**Main results**	**Model**	**Reference**
To develop a magnetic thermosensitive hydrogel for intravesical Bacillus Calmette-Guérin (BCG) delivery formulated with chitosan (CS), b-glycerophosphate (GP), and Fe_3_O_4_ magnetic nanoparticles (Fe_3_O_4_-MNP).	Magnetic chitosan hydrogel	Fe_3_O_4_-MNP	Solvent evaporation	186.2 nm	Positive: 38.4	89.73%	†A magnetic thermosensitive CS/GP hydrogel was a suitable matrix for extended BCG delivery by intravesical route. †The biodegradable and injectable thermosensitive gel showed a rapid solegel phase transformation. †Sustained delivery of BCG increased the antitumor efficacy and induced a high local immunity in bladder.	*In vivo* (Female Wistar rats-8-weeks old)	Zhang et al., 2013
To optimize and evaluate the antitumor efficacy of cationic chitosan (CS) nanoparticles encapsulating BCG for bladder tumor	Cationic chitosan (CS) nanoparticles encapsulating BCG	BCG-loaded CS	Data not shown	269–375 nm	Positive	42%	†CG-loaded chitosan nanoparticles resulted in increased survival rate. †Significant nanoparticle accumulation in bladder tissues was observed. †Cationic CS nanoparticles provide a significantly improved intravesical immunotherapy approach for bladder tumors.	*In vivo* (Rats)	Erdogar et al., 2014
To determine the direct effect of viable or heat-killed BCG and BCG cell wall skeleton (BCG-CWS) on UC cells *In vitro*	BCG Cell Wall Skeleton (BCG-CWS)	SMP105 BCG-CWS	Preparation of cell wall skeleton (CWS) (SMP-105) according Azuma et al. (1974) and Uenishi et al. (2007)	4.7 to 67.8 μm	Data not shown	Data not shown	†BCG induced cell growth retardation in highly malignant UC expressing integrin α5β1 (VLA5), suggesting VLA5 may be a biomarker of UC with sensitivity to BCG. †BCG-CWS is a promising substance which might replace BCG, preventing complications of viable BCG treatment.	*In vitro* (bladder cancer cell lines T24, HT1376, and RT4)	Kato et al., 2010
To evaluate of the ability of natural killer cells to cytolyze bladder cancer cells modified by R8-liposome-bacillus Calmette-Guérin (BCG)-cell wall skeleton (CWS) treatment	BCG Cell Wall Skeleton (BCG-CWS)	R8-liposome-bacillus Calmette-Guéin (BCG)-cell wall skeleton (CWS)	R8-liposome-BCG-CWS was prepared using a method described previously Homhuan et al. (2007)	Data not shown	Data not shown	Data not shown	†The induction of surface NKG2D ligands by R8-liposomeBCG-CWS rendered cancer cells more susceptible to cytolysis by lymphokine-activated killing. †T24 cells and RT-112 cells can directly respond to R8-liposome-BCG-CWS.	*In vitro* (T24 cells and RT-112 cells)	Miyazaki et al., 2011a
To determine if a non-living bacterial agent could be as efficacious as live BCG in a model of bladder cancer	BCG Cell Wall Skeleton (BCG-CWS)	R8-liposome-BCG-CWS	R8-liposome-BCG-CWS was prepared using a method described previously Homhuan et al. (2007)	Data not shown	Data not shown	Data not shown	†Rats receiving R8-liposome-BCG-CWS intravesically developed significantly fewer tumors. †R8-liposomeBCG-CWS significantly inhibited rat bladder carcinogenesis.	*In vivo* (8-week-old male Fisher-344 rats)	Miyazaki et al., 2011b
To develop a cell wall (CW) preparation from heat-killed bacillus Calmette-Guérin (BCG-CW) incorporated into octaarginine-modified cationized liposomes and to evaluate its immunoprotective potentiation in mice.	BCG Cell Wall Skeleton (BCG-CWS)	R8-liposome-BCG-CW	The CW fraction was prepared from heat-killed *Mycobacterium bovis* BCG Tokyo 172 cells according Joraku et al., 2008	232 to 270 nm	Positive: 19.9 to 26.9	Data not shown	†Confocal laser scanning microscopy showed enhanced incorporation of R8-liposome-BCG-CW into MBT-2 cells after 1 h of co-incubation. †0.1 mg R8-liposome-BCG-CW completely inhibited the growth of MBT-2 tumors while 0.1mg BCG-CW alone did not.	*In vitro* (bladder cancer cell line - MBT-2) and *In vivo* (female C3H/HeN mice - 7-weeks-old)	Joraku et al., 2008
To investigate the role of bladder cancer cells and DCs in internalization of BCG-CWS and initiation of the antibladder tumor effect using CWS-NP	Nanoparticle encapsulating BCG-CWS	CWS-NP	CWS-NP was prepared by the LEEL method described by Nakamura et al., 2014a	173 ± 8 nm	Positive: 41 ± 1	Data not shown	†Immune responses caused by the internalization of BCG-CWS by bladder cancer cells. †Tumor growth was significantly inhibited in mice that had been inoculated with mouse bladder cancer (MBT-2) cells containing internalized BCG-CWS.	*In vitro* (bladder cancer cell line - MBT-2) and *In vivo* (C3H/HeN mice (female, 8–10 weeks)	Nakamura et al., 2014a
To develop a novel packaging method that permits BCG-CWS to be encapsulated into lipid particles, as well as evaluate uptake efficiency of CWS-NP by mouse bladder tumor (MBT-2) cells *In vitro* and resulting tumor growth inhibition in mice bearing MBT-2 tumors	BCG-CWS encapsulated into lipid particles	CWS-NP	Preparation of CWS-NP by the LEEL method and the hydration methodh by Nakamura et al., 2014a	The diameter and zeta-potential of R8-Lip were 283 ± 16 nm and the diameter of CWS-NP/LEEL were 166 ± 2 nm.	The zeta-potential of R8-lip were 48 ± 2 and the zeta-potential of CWS-NP/LEEL 31 ± 0.4	The encapsulating ratio of BCG-CWS in the CWS-NP/LEEL was 57 ± 2%.	†CWS-NP was efficiently taken up by mouse bladder tumor (MBT-2) cells *In vitro* and inhibited tumor growth in mice bearing MBT-2 tumors. †Intravesically administered CWS-NP showed significant antitumor effects in a rat model presenting with naturally developed bladder tumors.	*In vitro (Bladder cancer cells - MBT-2) and In vivo (*Female C3H/HeN mice−7 weeks old)	Nakamura et al., 2014b

Cationic Chitosan (CS) NPs encapsulating BCG developed with the purpose of increasing the antitumor efficacy of BCG following intravesical administration have demonstrated significant advantages for the treatment of BC (Erdogar et al., 2014). Application of magnetic thermosensitive hydrogel developed with chitosan has been shown to be effective in increasing exposure of the bladder to BCG in addition to potentializing its immunological response (Zhang et al., 2013).

With the goal of overcoming side effects resulting from the administration of viable BCG, BCG cell wall skeleton (BCG-CWS) used as an immunomodulator in cancer patients (Uenishi et al., 2007, 2009; Hayashi et al., 2009) was shown to effectively control cell proliferation in UC, representing an efficient and safe alternative to BC immunotherapy (Kato et al., 2010). Nakamura et al. (2014a) was the first to develop a nanoencapsulated BCG-CWS (CWS-NP) using a liposome evaporated emulsified lipid (LEEL) method, resulting in a strong antitumor effect against MBT-2 BC cells as well as *in vivo* induced tumors (Nakamura et al., 2014a). CWS-NP was also shown to produce significant antitumor effects through internalization of BCG-CWS in BC cells, which contributes to the initiation of antitumor immunological activity (Nakamura et al., 2014b).

Another nanotechnological approach applied to BCG therapy consists of the use of modified nanoparticles incorporating BCG cell wall (BCG-CW) or skeleton. Utilization of octaarginine-modified liposomes incorporating BCG-CW (R8-liposome-BCG-CW) results in increased immunotherapeutic potential of BCG-CW for NMIBC through cellular internalization resulting in growth inhibition *in vivo* (Joraku et al., 2008). R8-liposome-BCG-CWS has also be used to investigate the suppressive effects of liposomes using a rat N-butyl-N-(4-hydroxybutyl) nitrosamine (BBN) induced BC model. This approach demonstrated that R8-liposome-BCG-CWS displays inhibitory effects against CD *in vivo* (Miyazaki et al., 2011b). Using this same approach, other studies have demonstrated that R8-liposome-BCG-CWS treatment results in induction of surface specific ligands (NKG2D) in BC cells, making them more susceptible to lymphokine-activated killing (LAK), indicating these cells are affected by R8-liposome BCG-CWS administration (Miyazaki et al., 2011a). These results demonstrate the efficiency of nanotechnology applications for optimization and development of novel BCG immunotherapy approaches for BC.

## Perspective

**Figure 1:**
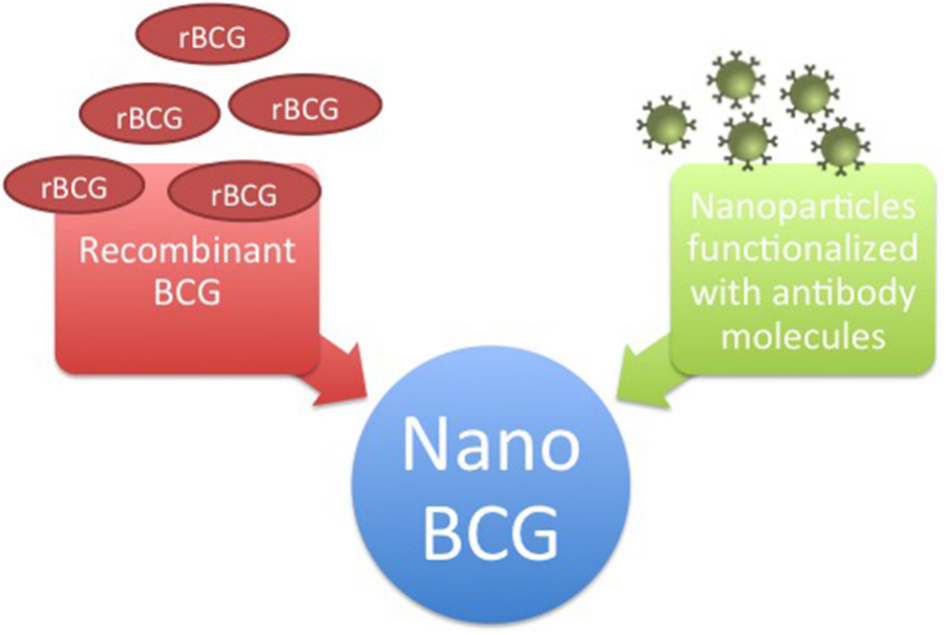
Combination of therapeutic approaches for bladder cancer treatment. Nano BCG has gain of effect by combination with recombinant BCG or by functionalization of nanoparticles with antibodies molecules.

Considering the promising results demonstrated by utilizing nanotechnology to develop new BC therapies, including success application of those approaches to BCG therapy, we believe that nanotechnology will provide significant advances for improving BC treatment. In this review, we described the main advances and applications of nanotechnology tools for development of novel treatments against BC, providing evidence that nanotechnology has contributed greatly to this effort by controlling drug release for longer periods, enabling the encapsulation of multiple drugs simultaneously, decreasing side effects, and increasing bioavailability. Nevertheless, we believe that combination therapies show more promise for effective treatment of this complex disease than individual approaches (Figure 1).

Although nanotechnology has resulted in significant progress for BCG immunotherapy, including increasing immunotherapeutic effects, prolonging exposure of the bladder tissue, and reducing adverse side effects, we believe this approach can be further enhanced through intravesical BCG delivery using NPs functionalized with antibody molecules against highly expressed receptors on the surface of BC cells such as EGFR to further target drug delivery and avoid systemic exposure and clearance of NPs from the body. Therefore, considering the emerging and motivating results using these approaches to treat BC, we believe that BCG delivery using NPs functionalized with monoclonal antibodies, in particular anti-EGFR, will provide a significantly improved delivery system for treatment of human BC.

## Author contributions

JB drafted the manuscript. CB wrote the introduction section, KB wrote the bladder cancer treatments section, and all authors, mainly AP, SG, TC, FS, and KB were responsible for conception, manuscript review, and critical intellectual input.

### Conflict of interest statement

The authors declare that the research was conducted in the absence of any commercial or financial relationships that could be construed as a potential conflict of interest.
